# Reproducibility challenges in robotic surgery

**DOI:** 10.3389/frobt.2023.1127972

**Published:** 2023-03-15

**Authors:** Angela Faragasso, Fabio Bonsignorio

**Affiliations:** ^1^ The Service Robotics Laboratory, Department of Precision Engineering, School of Engineering, The University of Tokyo, Tokyo, Japan; ^2^ ERA Chair in AI for Robotics, Head of AIFORS Lab FER, University of Zagreb, Zagreb, Croatia

**Keywords:** reproducibility, surgical robotics, medical robotics, translational medical robotics, experimental methodology, scientometrics

## Abstract

Reproducibility of results is, in all research fields, the cornerstone of the scientific method and the minimum standard for assessing the value of scientific claims and conclusions drawn by other scientists. It requires a systematic approach and accurate description of the experimental procedure and data analysis, which allows other scientists to follow the steps described in the published work and obtain the “same results.” In general and in different research contexts with “same” results, we mean different things. It can be almost identical measures in a fully deterministic experiment or “validation of a hypothesis” or statistically similar results in a non-deterministic context. Unfortunately, it has been shown by systematic meta-analysis studies that many findings in fields like psychology, sociology, medicine, and economics do not hold up when other researchers try to replicate them. Many scientific fields are experiencing what is generally referred to as a “reproducibility crisis,” which undermines the trust in published results, imposes a thorough revision of the methodology in scientific research, and makes progress difficult. In general, the reproducibility of experiments is not a mainstream practice in artificial intelligence and robotics research. Surgical robotics is no exception. There is a need for developing new tools and putting in place a community effort to allow the transition to more reproducible research and hence faster progress in research. Reproducibility, replicability, and benchmarking (operational procedures for the assessment and comparison of research results) are made more complex for medical robotics and surgical systems, due to patenting, safety, and ethical issues. In this review paper, we selected 10 relevant published manuscripts on surgical robotics to analyze their clinical applicability and underline the problems related to reproducibility of the reported experiments, with the aim of finding possible solutions to the challenges that limit the translation of many scientific research studies into real-world applications and slow down research progress.

## 1 Introduction

Reproducibility and replicability are the means for gaining confidence in scientific results, and they are the main features that allow to consider “scientific” experiment results. However, despite all the scientific efforts and research publications, more than 70% of researchers have tried and failed to reproduce another scientist’s experiments, and more have failed to reproduce their own experiments (Baker, 2016). These failures demonstrate that there is a reproducibility crisis, one of the most important issues of scientific enterprise in our times, which is mainly due to the pressure to publish and selective reporting. In the artificial intelligence and robotics fields, the situation is made more difficult by the fact that good reproducibility practices are still not mainstream. The solution to this problem is linked to open research where scientists share all the processes, provide accurate and clear documentation, and estimate the uncertainty inherent in their results and inferences, as reported in the reproducibility spectrum derived by Peng (2011). Moreover, efforts from academic institutions, scientific journals, funding organizations, and conference organizers are needed to overcome this crisis, which is affecting many fields, especially robotics (Bonsignorio and Del Pobil, 2015; Bonsignorio, 2017).

Benchmarking is a community-driven activity that allows performance evaluation under controlled conditions involving consensus-based decisions on how to make reproducible, fair, and relevant assessments (Bligaard et al., 2016). It enables the comparison of different systems in common, predefined settings and provides a set of metrics together with a proper interpretation to perform an objective evaluation and hence test reproducibility and replicability of scientific discoveries. Reproducibility, replicability, and benchmarking are crucial in medical robotics, but due to patenting, safety, and ethical issues, they get more complex than in any other robotics-related fields (Sharkey and Sharkey, 2012; Leenes et al., 2017; Yang et al., 2017).

### 1.1 Robotics surgery

Robots in the medical field are revolutionizing surgery, diagnosis, treatment, care, and logistics. Mechanical robotic systems enhanced with sophisticated software are nowadays employed in surgical robotics, micro-robotics, prosthetic-rehabilitation, and hospital-ambulatory care (Dupont et al., 2021). They improve the accuracy of the surgical tasks, allow remote treatment, provide precise and objective diagnosis, and offer at-home support. In the operating room, robots not only assist the surgeon in performing the procedure but also provide superhuman capabilities. In minimally invasive robotic surgery, the surgeon manipulates robotic instruments, which are inserted into the human body through trocars, *via* a control panel, and views the anatomical area of interest in a 2D screen (Bonjer, 2017). Minimally invasive surgery has brought many benefits for patients, such as less trauma to muscles, nerves, and tissues; less bleeding, pain, and scarring; and shorter hospital stays, but it is much more complex for surgeons who need special training to manipulate the devices and have limited haptic and visual feedback, which are instead available in traditional open surgery (Lajkó et al., 2021; Rahimli et al., 2022).

To improve the outcome of surgical operations and experience of medical practitioners, researchers have been developing different surgical instruments, robotics platforms, sensing devices, and software. However, most of these discoveries were not able to overcome the “valley of death” which is the place between the lab bench and the marketplace where many good biomedical ideas wither away and die. Although translational research is a crucial step for discovering new treatments and improving healthcare systems, translating early discoveries into effective treatments for patients is time-consuming, expensive, and often unsuccessful—the rate of success in translational science is less than 1% (Getz et al., 2020).

In this review paper, the reproducibility of 10 of the most relevant experimental papers on “surgical robotics” are analyzed from a methodological point of view, following a general set of guidelines for manuscripts involving experiments. The papers have been selected on the basis of scientometric criteria, which is one of the most common methods for the analysis of scientific products and patents employing indexes that reflect the impact of a study. In this study, we focus only on reproducibility, which together with replicability are the basic requisites of a scientific experiment are essential for performance evaluation and prerequisites for benchmarking. Criteria for benchmarking assessments will be performed in future analysis.

The aim of this study is to underline common practices that limit the replicability of published works and help define rigorous experimental methodologies for surgical robotics research to ensure robust and unbiased experimental design, methodology, analysis, interpretation, and reporting of results.

This paper is organized as follows: the criteria for experimental evaluation and data selection are described in Section 2. Results of the derived analysis are shown in Section 3. Conclusions and discussion are given in Section 4. Limitation of the proposed analysis are reported in Section 5.

## 2 Materials and methods

**FIGURE 1:**
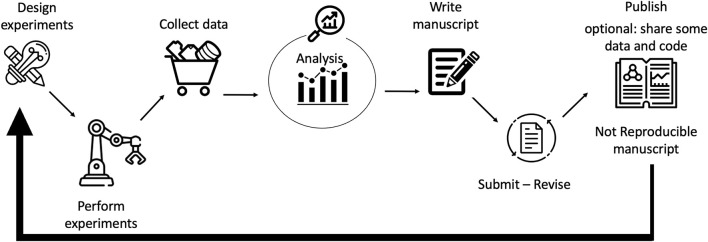
Not-reproducible research lifecycle: from designed and executed experiments, collection and analysis of data are performed from which a manuscript is written, submitted, revised, and eventually published. Optionally, together with the manuscript, data and source code are also made available. From the results obtained, a new hypothesis and experiments can be drawn. It is not possible to reproduce the experiments described in the published paper.

**FIGURE 2:**
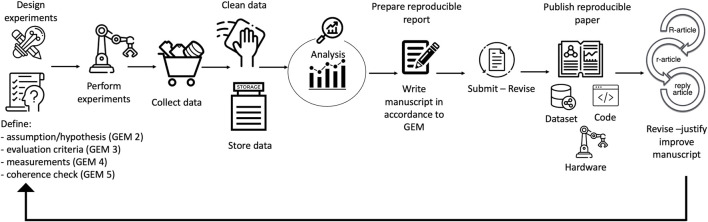
Reproducible research lifecycle: to have a fully reproducible paper, when the experiments are designed in accordance with the GEM guidelines, it is important to respect the following criteria. GEM 2: Are the system assumptions/hypotheses clear? GEM 3: Are the evaluation criteria spelled out explicitly? GEM 4: What is being measured and how? The claims based on the experiment and performance evaluations should be supported by measurable quantities. GEM 5: Do the methods and measurements match the criteria? After those steps, experiments are performed, and data are collected, cleaned, stored, and analyzed. The written manuscript should follow the GEM guidelines and have a clear and detailed description of all the information needed to reproduce the presented work. It is important to share, together with the paper, all the information necessary to reproduce the experimental work, i.e., the dataset, source code, and details about the hardware. The published reproducible experimental paper (R-article) will follow a new lifecycle and be eventually reproduced by other researchers in a replication article (r-article). The authors of the original R-article can then reply to the r-article with a reply article (Bonsignorio, 2017). From the published papers, new experiments can be designed and defined.

Generally, published scientific research follows the lifecycle shown in Figure 1, i.e., from designed and executed experiments, collection and analysis of data are performed, from which a manuscript is written, submitted, revised, and eventually published. Optionally, together with the manuscript, data and the source code are also made available. From the results obtained, new hypotheses and experiments can be drawn. Unfortunately, there are complications which limit the reproducibility and replicability of those manuscripts that are related to technological advances (sometimes there are complex and huge data, limitations in the computational power, and a lack of ability or will to share the discovery) and human errors (poor reporting and flawed analysis). However, by adding a few more steps to this cycle and following the good experimental methodology (GEM) guidelines described further, reproducible research can be performed and reported, as shown in Figure 2. In particular, to obtain a fully reproducible experimental paper, when the experiments are designed, the assumptions, evaluation criteria, measurements, and coherence check should be defined in detail. After the experiments are performed, a plan for data storage (data management plan), collection, and cleaning of the data through version control must be carried out before analyzing the collected data. Additionally, when writing the manuscript, it is important to share, together with the paper, all the information necessary to reproduce the experimental work, i.e., the dataset, source code, and details about the hardware. The published reproducible experimental paper (R-article) will follow a new lifecycle and will be eventually reproduced by other researchers in a replication article (r-article). The authors of the original R-article can then reply to the r-article with a reply article (Bonsignorio, 2017). From the published papers, new experiments can be designed and defined.

Most robotic systems involve practical experimentation, which must be carried out efficiently and reported properly. Hence, it is essential to define a set of metrics that can help identify high-quality manuscripts of replicable experimental work. In the following sections, the metric used to evaluate the analyzed manuscripts and the selection process are described in detail.

### 2.1 Guidelines for experimental robotics papers

Based on the general guidelines for robotics papers involving experiments, the following set of eight questions can be used to identify high-quality reporting of reproducible experimental work (Bonsignorio et al., 2007):

1. Is it an experimental paper?
     An experimental paper is a manuscript whose results, conclusions, and discussions exclusively and largely depend on experimental work.
2. Are the system assumptions/hypotheses clear?
     The assumption, hypothesis, and system limits should be properly reported.
3. Are the evaluation criteria spelled out explicitly?
     The paper should address a relevant research problem and detail performance metrics and evaluation.
4. What is being measured and how?
     The claims based on the experiment and performance evaluations should be supported by measurable quantities. The data types and physical meaning of the measurements should be clear: numerical, categorical (e.g., yes/no), or ordinal (rankings).
5. Do the methods and measurements match the criteria?
     The measurement method should be properly and operationally described, and the measured data should match the desired criteria.
6. Is there enough information to reproduce the work?
     There should be enough information which will allow independent researchers to repeat the work.
7. Do the results obtained give a fair and realistic picture of the system being studied?
     The experimental setting should be realistic, and factors that can affect the performance should be properly identified and controlled.
8. Are the drawn conclusions precise and valid?
     The conclusions drawn should be consistent with the research questions the manuscript tackles, the assumptions, and the evaluation criteria.

If the answer to all those questions is “yes,” then the analyzed paper results will be fully reproducible. Those criteria represent the GEM guidelines, which are valid for any experimental work, and will be used in the following section for the evaluation of manuscripts related to surgical robotics.

### 2.2 Clinical applicability of surgical robotics research

Clinical applicability can be defined as the extent to which the results observed in published studies are likely to reflect the expected outcomes when intervention is applied to real-world conditions. A series of good clinical research practices (GCPs) define the validity of medical research and provide guidance for their implementations (World Health Organization, 2005). A new drug, for instance, should undergo preclinical studies (*in vitro*, *in vivo*, and *ex vivo*) before a clinical trial is approved. As for any other medical research, including surgical robotics, it is important to carry out experimental evaluations on environments and systems that are as close as possible to the clinical evaluation. For this reason, it is desirable, although not essential, to conduct cadaver or animal tests. An alternative to cadaver and animal experimental tests, which can closely reproduce the characteristic of the operating setting, is the use of phantoms, which try to replicate the clinical conditions as much as possible. Even though this aspect is not strictly correlated to the reproducibility of the research, it is fundamental to assess the technology readiness level (TRL), which represents the maturity stage of the proposed solution. To evaluate the clinical applicability of the research performed in the selected manuscripts, the following criteria are added to the general guidelines:

9. Is the experimental validation replicating the clinical settings?
     The experiments should be performed on systems which replicate with confidence the clinical settings in which the proposed solution should operate.

We underline that, in general, the authors of very early-stage research (low TRL) may not perform experimental testing on cadavers or animals, although this will be a prerequisite for clinical translation.

### 2.3 Data selection

This section outlines some interesting trends emerging from published papers on surgical robotics. It provides only a general overview and not an exhaustive survey in the field.

Scientometrics, i.e., the extrapolation of indexes which provide objective quantitative data that reflect the impact of study, research, or institution, has been applied in many research fields, including robotic surgery (Dupont et al., 2021; Vidal et al., 2022; Zhang et al., 2022; Zhou and Li, 2022). Web of Science Core Collection (WoSCC) is a trusted citation index for locating research across a curated, multidisciplinary set of journals, books, and conferences and widely used in scientometric studies. The selection of the manuscripts analyzed in the following section has been carried out following scientometric analysis criteria. The Science Citation Index Expanded of WoSCC, accessed on 20 October 2022, was used to collect data from published original articles in robotic surgery. The search selected manuscripts published between 1 October 2012 and 1 October 2022 on topics related to surgical robotics that used “surgical robot” as a keyword. The papers were sorted by the highest citation index and evaluated following the guidelines for robotics research involving experiments summarized previously.

The data were extracted as an Excel file and analyzed. The search gave 332 results with an average of 9.12 citations per item and an h-index of 27. Table 1 reports the corresponding author, title of the paper, and place of publication of the 10 most cited manuscripts. Review papers and surveys, as well as papers that are not experimental (the answer to question 1 is “no”), were excluded from the list as they are not relevant for the study conducted in this manuscript. We have restricted our analysis to the top 10 most cited articles. The reproducibility of the analyzed manuscripts was evaluated considering the GEM guidelines. For each experimental paper, we examined the assumptions, hypotheses, evaluation criteria, measurements, methods, conclusions, and information (software, descriptions, and data) essential for the reproduction of the described experiments. Additionally, we also analyzed the clinical applicability of the proposed solutions in correlation with the environments in which they are supposed to operate.

**TABLE 1:** Scientometric extraction of surgical robotics papers based on the Citation Index Expanded of WoSCC.

Authors (Year)	Title	Published in
Xu et al. (2014)	Development of the SJTU Unfoldable Robotic System (SURS) for Single Port Laparoscopy	IEEE/ASME Transactions on Mechatronics
Do et al. (2014a)	Hysteresis modeling and position control of tendon-sheath mechanism in flexible endoscopic systems	Mechatronics
Chen et al. (2013)	Inverse transmission model and compensation control of a single-tendon–sheath actuator	IEEE Transactions on Industrial Electronics
Do et al. (2014b)	An investigation of friction-based tendon sheath model appropriate for control purposes	Mechanical Systems and Signal Processing
Roy et al. (2016)	Modeling and estimation of friction, extension, and coupling effects in multisegment continuum robots	IEEE/ASME Transactions on Mechatronics
Shin and Kwon (2013)	Surgical robot system for single-port surgery with novel joint mechanism	IEEE Transactions on Biomedical Engineering
Kim et al. (2018)	Sensorized surgical forceps for robotic-assisted, minimally invasive surgery	IEEE Transactions on Industrial Electronics
Li et al. (2015)	A novel tele-operated flexible robot targeted for minimally invasive robotic surgery	Engineering
Mitsuishi et al. (2013)	Master–slave robotic platform and its feasibility study for micro-neurosurgery	The International Journal of Medical Robotics and Computer Assisted Surgery
Su et al. (2018)	Safety-enhanced collaborative framework for tele-operated minimally invasive surgery using a 7-DoF torque-controlled robot	International Journal of Control, Automation and Systems

## 3 Data analysis

The most cited experimental paper by Xu et al. (2014) presents the SJTU Unfoldable Robotic System (SURS) for single-port laparoscopy. The robotic system can fit through a hole of 12 mm diameter and is composed of a dual-arm system and 3D visual guidance. Experimental evaluation is carried out to test the mechanism deployment during insertion, payload capabilities, and efficiency during teleoperation.

For what concerns criterion 4, experiments regarding the deployment of the system, bending error before and after compensation, payload tests, and performance of the robotic device during teleoperation are presented. A numerical evaluation of the payload and bending errors and the categorical evaluation of the ability to perform suturing and peeling tasks during teleoperation were conducted. Although the manuscript presents a rigorous description of the design and modeling of the system, criteria 5, 6, 8, and 9 are not satisfied as some of the experiments performed do not have a clear evaluation of the metric and there is not enough information provided by the authors that allows other researchers to reproduce this work. In particular, the deployment of the robotic system is performed in “open environments”; hence, it is not possible to evaluate its effectiveness in laparoscopic settings. Additionally, the conclusion drawn, especially in the teleoperation tests, is not supported by a methodological experimental valuation. For the execution of the tasks, snapshots of the system performing suturing tasks and grape peeling are reported; however, there is no quantitative evaluation of those experiments. The clinical settings in which this robot should operate are not properly or even closely reproduced, and details about the teleoperation tasks (subject controlling the robot, number of trials, time, *etc.*) are also missing.

A novel approach to modeling and controlling of a tendon–sheath mechanism for endoscopic applications is derived in the paper by Do et al. (2014a). The proposed solution can identify the non-linear backlash phenomena, regardless of the curvature and sheath angles. Experimental tests are conducted to prove the validity and performance of the derived model and control scheme. Numerical data, backlash hysteresis non-linearity, and the error of the derived model are the results of the experimental validation (criteria 4). The authors clearly defined the evaluation criteria and measurements employed, limitations, and assumptions of their approach. The results provide a realistic picture of the system, and the conclusion is precise and valid. However, there are not enough details which will allow other researchers to reproduce the same system and validate the derived results; hence, criterion 6 is not satisfied. Moreover, the experimental settings do not replicate the clinical setup in which the system should operate (criterion 9 is not satisfied).

Modeling and compensation of single-tendon–sheath actuators are the objectives of the work proposed by Chen et al. (2013). A displacement transmission model for a single-tendon–sheath transmission system based on the force transmission model and a novel control strategy for the distal-end force and position is proposed. Experimental validation is conducted to investigate the validity of the derived model. In the experiment, the system is evaluated under position control and force control modes, and numerical quantities, i.e., distal displacement, distal force, and tracking errors, are measured (criterion 4). The derived model is properly reported, and the assumptions and limitations are underlined; however, the experimental setup is poorly described. Moreover, there is no comparison with other state-of-the-art methodologies, and there is not enough information provided for the full reproducibility of this work (criterion 6 is not satisfied). Additionally, the validation is performed on an experimental setting which does not reproduce the clinical environment in which the system should work, and hence criterion 9 is not satisfied.

The modeling of a tendon–sheath mechanism is also the focus of the work reported by Do et al. (2014b). The proposed solution allows the characterization of the friction lag and hysteresis in the presliding and sliding regimes in arbitrary configurations of the sheath. Rigorous mathematical derivation is reported, and experimental validation is performed in simulation with real hardware. For criterion 4, the measurement is based on numerical data, friction force, and transmission error. The limitations and assumptions are clearly stated; however, also in this work, there is not enough information that will allow independent researchers to repeat the same experiments and validate the proposed solution (criterion 6 is not satisfied). Additionally, as the experimental setting does not reproduce the clinical setting where the system should operate, criterion 9 is also not satisfied.

Roy et al. (2016) proposed a model-based estimation and actuation compensation framework for continuum robots, enabling the online estimation of modeling uncertainties and hence adaptability to different environment conditions and friction. The problem setting, assumptions, and model description are clearly stated. The mathematical derivation is rigorous and clear. The numerical error, friction, and position accuracy are the numerical quantities evaluated in this work (criterion 4). In the manuscript, the experimental setup is poorly described, and there is not enough information which will allow researchers to reproduce the results presented (criterion 6 is not satisfied). Moreover, as the experimental settings are not even closely similar to the clinical setup, criterion 9 is not satisfied.

A novel joint mechanism for single-port surgery, which can prevent hysteresis and achieve accurate motion with a large force, is developed by Shin and Kwon (2013). The design of the robotic system and the kinematics of the mechanism are reported. Preliminary tests are performed to validate the proposed solution. Analysis of the workspace, hysteresis, and tool tip force are the numerical data measured. Categorical measurement is performed to evaluate the performance of the system on executing a laparoscopic task (criterion 4). In this work, criteria 5, 6, 7, 8, and 9 are not satisfied; the experimental setup and the details of the overall device are poorly represented. Hence, it is not possible to reproduce the system presented in this manuscript. In the block transfer task, in which five volunteers performed the task and the average time was measured, characteristics of the desired features in the execution of the task are missing. The authors claim that the average time for the block transfer task was long because of the intuitiveness of the master interface; however, there are no accurate details about the interface, and it is not clear why a different interface was not used. Details about the volunteer experimental settings and the comparison with other state-of-the-art systems are also omitted.

Kim et al. (2018) presented a sensorized surgical forceps for robotic-assisted, minimally invasive surgery with five degrees of freedom of force/torque-sensing capabilities. The miniaturized sensor can be realized at low cost, and it is disposable and adaptable to many configurations. Calibration, mathematical derivation, and experimental tests using a surgical robot have been conducted to prove the validity of the proposed device. The evaluation criteria and limitations are clearly stated and properly evaluated in the manuscript. Regarding criterion 4, numerical quantities, exerted force, and torque are being measured in the experiments. Criterion 6 is not satisfied as the details of the experimental tests are missing; hence, it is impossible for another researcher to reproduce the same system and evaluate the results. Moreover, the method and measurements do not properly match the criteria. There is no evaluation of different configurations of surgical forceps (criterion 5 is not satisfied). Additionally, the results obtained do not give a fair and realistic picture, and the conclusions drawn are not precise. In particular, in the grasping experiment on the “tissue,” it is difficult to understand the position of the tissue during the tests, and justification on the type of material is omitted (criteria 7 and 8 are not satisfied). Criterion 9 is also not satisfied as the experimental setting is far from the clinical setting in which the device is supposed to be used.

A novel flexible robot system with a constrained tendon-driven serpentine manipulator (CTSM) is presented in the manuscript by Li et al. (2015). The design of the serpentine manipulator, teleoperation scheme, and kinematic model are first reported. Simulation is performed for the workspace and dexterity comparison. After fabrication, experiments are conducted to prove the validity of the device on target approaching and weight-lifting tasks. For what concerns criterion 4, the numerical quantities measured are the workspace of the manipulator and dexterity. Additionally, the system ability to execute a nasal cavity-exploring task is also evaluated (categorical measurement). The manuscript is very easy to follow and clear. Assumptions and limitations are properly reported. However, there is not enough information which will allow other researchers to reproduce the same work (criterion 6 is not satisfied). It has to be noticed that this is the first paper in which experimental evaluations using phantoms that mimic the human’s nasal cavity are performed.

Mitsuishi et al. (2013) dealt with a problem related to neurosurgery by proposing a novel master–slave robotic platform which enhances the positioning accuracy and allows for smooth trajectory generation. The aim is to create a system that can perform complicated surgical tasks such as anastomosis with high accuracy. The details about the platform, working principle, and control system are clearly reported. Experimental tests have been performed to evaluate the performance of the proposed robotic system on the execution of pointing, tracing, and anastomosis tasks. The numerical measurements consider the execution time and error comparison between manual and robotic operations. The categorical measurement is related to the ability to execute different tasks (criterion 4). The limitations of the proposed solution, which are mainly related to the completion time, are extensively discussed. Compared to other manuscripts, which also performed categorical evaluation, there are more tests that involve more than two subjects and also an expert surgeon in this work. However, full reproducibility is not possible; hence, also, for this work, criterion 6 is not satisfied.

A safety-enhanced collaborative framework using a redundant robot is realized in the last paper of the 10 most cited papers (Su et al., 2018). A Cartesian compliance strategy and a null-space strategy are combined to allow flexibility and safe solution in the operating room. Event-based procedures are interchanged during the surgical tasks, and the virtual reality interface is implemented for online visualization of minimally invasive procedures. The dynamic and kinematic models of the redundant manipulator and the implemented teleoperation system are reported in detail. The experimental test is conducted to evaluate the validity of the proposed solution. For criterion 4, the numerical quantities measured are the error and Cartesian accuracy. A categorical measurement of the execution of a tracking task is also performed. As there is not enough information to reproduce this work and validation is not performed in a proper clinical mock-up, criteria 6 and 9 are not satisfied in this work.

Table 2 summarizes the results of this analysis. Although all 10 papers are exceptionally good, we can notice that none of them is fully reproducible, and, most surprisingly, none of them reports experimental tests with animals or cadavers. Moreover, only two of them performed evaluations using a phantom that mimics the characteristics of the human body.

**TABLE 2:** Manuscript analysis based on the nine criteria for experimental surgical robotics papers.

Criteria	Xu et al. (2014)	Do et al. (2014a)	Chen et al. (2013)	Do et al. (2014b)	Roy et al. (2016)	Shin and Kwon (2013)	Kim et al. (2018)	Li et al. (2015)	Mitsuishi et al. (2013)	Su et al. (2018)
										
1. Is it an experimental paper?	*✓*	*✓*	*✓*	*✓*	*✓*	*✓*	*✓*	*✓*	*✓*	*✓*
2. Are the system assumptions and hypotheses clear?	*✓*	*✓*	*✓*	*✓*	*✓*	*✓*	×	*✓*	*✓*	*✓*
3. Are the evaluation criteria spelled out explicitly?	*✓*	*✓*	*✓*	*✓*	*✓*	*✓*	*✓*	*✓*	*✓*	*✓*
4. What is being measured and how?	Numerical and categorical	Numerical	Numerical	Numerical	Numerical	Numerical and categorical	Numerical	Numerical and categorical	Numerical and categorical	Numerical and categorical
5. Do the methods and measurements match the criteria?	×	*✓*	*✓*	*✓*	*✓*	×	×	*✓*	*✓*	*✓*
6. Is there enough information to reproduce the work?	×	×	×	×	×	×	×	×	×	×
7. Do the results obtained give a fair and realistic picture?	*✓*	*✓*	*✓*	*✓*	*✓*	×	×	*✓*	*✓*	*✓*
8. Are the conclusions drawn precise and valid?	×	*✓*	*✓*	*✓*	*✓*	×	×	*✓*	*✓*	*✓*
9. Is the experimental validation replicating the clinical settings?	×	×	×	×	×	×	×	*✓*	*✓*	×

## 4 Discussion

The hardest aspect to accomplish when writing an experimental paper is related to criterion 6 as the effectiveness of an approach is correlated with the details necessary to reproduce the results. The lack of information attached to the manuscript is what makes this point very critical. In general, if the manuscripts concern experiments that are performed in simulations, then the simulator should be made available together with the source code and setup details. If it is not possible to share the simulator, then there should be enough details to implement the system on a different platform and obtain comparable results. If the experiments are performed on real hardware, then the description of the experiential settings, source code, and any other information which may affect the results should be provided. In particular, for surgical robotics research, if the system is used by subjects, details about the subjects, trials, and any other information that may affect the results should also be described in detail. If a new component is realized, then accurate information about the design, such as the CAD model, should also be made available. The details on the machine in which the algorithms have been tested should be made available.

As reported in Section 2.3, papers that were not experimental were discarded from this analysis. We, however, have to acknowledge that the most cited paper resulted from the selection was “Raven-II: An Open Platform for Surgical Robotics Research” by Hannaford et al. (2012), which was discarded because it was not experimental. In this manuscript, an open-source surgical platform for collaborative research is described. This is an important work for the robotics surgical community because it allowed researchers from different universities to work on the same surgical platform and have comparable results. Open-source robotics, i.e., open-source hardware and software, is the key to fast improvements in robotics research and can potentially help solve some of the problems related to criterion 6. Other open platform and software, such as the da Vinci Research Kit (dVRK) (Kazanzides et al., 2014), the Robot Operating System (ROS) (Joseph, 2017), and the soft robotics toolkit (Holland et al., 2014), have been proven to be successful and great tools for the robotics community in the last decade. We hope that more systems and benchmarking platforms like Raven will be developed in the future and made available for the surgical robotic society.

It has to be noticed that many of the selected papers deal with modeling and controlling of tendon–sheath mechanisms. In fact, power transmission that can be delivered through a tendon–sheath mechanism has been extensively exploited in the last decade. It consists of an actuation cable (tendon) that is enclosed inside a hollow coil wire (sheath). The main feature of a tendon–sheath system is its ability to pass through a long narrow and tortuous path and operate in small areas. Although a tendon–sheath system has been used in many robotic applications, the non-linear characteristics of these systems attributed to the friction losses are not fully explored, leading to considerable difficulties in optimizing the system performance. They are very beneficial for the surgical system, which have to access the surgical site through narrow passages and have high dexterity to perform safe manipulation (Kim et al., 2022).

From this study, it is evident that experimental papers on surgical robotics are very often missing features that will allow independent researchers to reproduce the described work and compare the results. Although progress in this direction has been made, especially in a field like machine learning, there is still room for considerable improvement in intelligent robotics, especially surgical robotics. There is a need to overcome the anxiety that disclosing data will expose flaws and inconsistencies. The solution to this fundamental problem is correlated with the way we publish our research and can be solved by following different publication standards and implementing the R-article practice as, for example, in the IEEE RAS Robotics and Automation Magazine.

This study is not a criticism of the selected papers, which are, without any doubt, outstanding contributions, but aims at highlighting common practices which affect the way surgical robotics research is currently performed and reported. With the constructive criticism presented in this manuscript, we hope to raise the attention of our community toward this problem and overcome the barriers that consistently limit our evolution.

## 5 Limitation of the proposed analysis

It is important to notice that although scientometrics has been established as a good way to determine the impact of research and is widely used as a proxy for selecting relevant papers in different scientific fields, the selection of papers in this study does not represent the most interesting or best scientific work in surgical robotics, as we cannot relate this to the citation index. However, it provides, in our opinion, an appropriate method to sample a collection of relevant surgical robotics research.

We have to underline that the analysis conducted in this manuscript, although very rigorous, was performed only by the two authors. We invite the readers to conduct the same analysis, extrapolate the table related to the GEM guidelines, and comment on the following link: Reproducibility Table.

## Nomenclature

GEM: good experimental methodology
GCP: good clinical research practice
TRL: technology readiness level
RAS: Robotics and Automation Society
IEEE: Institute of Electrical and Electronics Engineers.
